# The trends of complementary alternative medicine use among cancer patients

**DOI:** 10.1186/s12906-021-03338-7

**Published:** 2021-06-08

**Authors:** Abdul Rahman Jazieh, Khadega A. Abuelgasim, Husam I. Ardah, Mohammad Alkaiyat, Omar B. Da’ar

**Affiliations:** 1 Department of Oncology, Ministry of National Guard Health Affairs, King Abdulaziz Medical City, P.O. Box 22490, Riyadh, 11426 Kingdom of Saudi Arabia; 2 Ministry of National Guard Health Affairs, King Saud bin Abdulaziz University for Health Sciences, P.O. Box 22490, Riyadh, 11426 Kingdom of Saudi Arabia; 3 King Abdullah International Medical Research Center, Ministry of National Guard Health Affairs, P.O. Box 22490, Riyadh, 11426 Kingdom of Saudi Arabia; 4 Department of Biostatistics and Bioinformatics, King Abdullah International Medical Research Center, Ministry of National Guard Health Affairs, Riyadh, Kingdom of Saudi Arabia; 5 Department of Health Systems, College of Public Health, Ministry of National Guard Health Affairs, King Saud bin Abdulaziz University for Health Sciences, Riyadh, Kingdom of Saudi Arabia

**Keywords:** Complementary and alternative medicine, CAM, Cancer patients, Trends

## Abstract

**Background:**

The use of complementary and alternative medicine (CAM) is common among cancer patients and it may reflect the individual and societal beliefs on cancer therapy. Our study aimed to evaluate the trends of CAM use among patients with cancer between 2006 and 2018.

**Methods:**

We included 2 Cohorts of patients with cancer who were recruited for Cohort 1 between 2006 and 2008 and for Cohort 2 between 2016 and 2018. The study is a cross-sectional study obtaining demographic and clinical information and inquiring about the types of CAM used, the reasons to use them and the perceived benefits. We compared the changes in the patterns of CAM use and other variables between the two cohorts.

**Results:**

A total of 1416 patients were included in the study, with 464 patients in Cohort 1 and 952 patients in Cohort 2. Patients in Cohort 2 used less CAM (78.9%) than Cohort 1 (96.8%). Cohort 1 was more likely to use CAM to treat cancer compared to Cohort 2 (84.4% vs. 73%, respectively, *p* < 0.0001,); while Cohort 2 used CAM for symptom management such as pain control and improving appetite among others. Disclosure of CAM use did not change significantly over time and remains low (31.6% in Cohort 1 and 35.7% for Cohort 2). However, physicians were more likely to express an opposing opinion against CAM use in Cohort 2 compared to Cohort 1 (48.7% vs. 19.1%, *p* < 0.001, respectively).

**Conclusion:**

There is a significant change in CAM use among cancer patients over the decade, which reflects major societal and cultural changes in this population. Further studies and interventions are needed to improve the disclosure to physicians and to improve other aspects of care to these patients.

## Background

The use of complementary and alternative medicine (CAM) is common in different cultures and among healthy individuals as well as patients with various ailments, especially chronic diseases particularly cancer [1–3]. National Centre for Complementary and Alternative Medicine (NCCAM) defines CAM as a group of various medical and health care systems, practices, and products that are not currently thought of as part of conventional medicine [4]. Pattern of CAM use differs according to the socioeconomic status, geography, various religious and spiritual backgrounds [4]. Patients with cancer are well known to use CAM for multiple reasons, driven by the seriousness and the life-threatening nature of the disease and the multiple complex medical psychosocial and emotional problems they are facing. Most cancer patients use alternative medicines as complementary means to help control symptoms. Smaller numbers of patients adopt alternative medicine treatments instead of mainstream therapy [5].

A recent systematic review of studies published between 2009 and 2018 reported about 51% of patients with cancer used CAM to treat the cancer, its complication or improve general health. Younger age, Female patients, higher education and income were characteristics associated with more use [6]. The reported prevalence of CAM use is higher than what reported in an earlier systematic review of studies conducted in 18 countries which also increase in the use over time and variation among countries with highest being in the US and lowest in Italy and Netherland [7].

It is critical to evaluate the CAM use in patients with cancer to understand their needs and help address them but also to prevent any harm form its use. The harms of CAM use may result from patients delaying or avoiding seeking proper cancer treatment leading to increase risk of cancer progression and reduce the chance of cure. On the other hands, CAM may interfere with treatment and reduce its efficacy leading to worse outcome. Furthermore, CAM may result in direct harm to the patients due to its toxicity or interaction with other medications leading to patients suffering in decline in quality of life [8–10]. In a large retrospective study of US National Cancer Database including 1,901,815 patients, the use of CAM was associated with refusal of conventional cancer treatment, and with a 2-fold greater risk of death compared with patients who had no complementary medicine use [11].

In Saudi Arabia, earlier studies reported the prevalent use of CAM among Saudi patients with cancer for different reasons varying from treatment and symptom relief to quality of life improvement. The reported CAM used by Saudi cancer patients includes dietary supplements and non-dietary supplement remedies [12]. Even though the use of CAM among cancer patients has some beneficial outcomes, it can lead to some potential risks such as interactions with chemotherapy drugs [13, 14].

These variations have been reported extensively in the past, however, data on trends and changes in CAM use overtime especially among our patients’ population do not exist. Therefore, we have conducted this study to compare CAM use among patients with cancer over 10 years.

## Methods

### Study design

A cross-sectional study included patients with cancer diagnosis served at the Oncology Department at King Abdulaziz Medical City of Ministry of National Guard Health Affairs, Riyadh, KSA. Ethical approvals for the studies were obtained from the Institutional Review Board at King Abdullah International Medical Research Center, Riyadh, Saudi Arabia. Participants consented before enrollment in the study.

### Inclusion criteria

Inclusion criteria allowed enrollment of any patient with a diagnosis of malignancy who is willing to participate in the study and complete the questionnaire.

### Tool description

The tool was a survey questionnaire written in a simple fashion and the research coordinator was available to help, in order to ensure that the majority of patients will be able to complete the survey without any difficulties. The questionnaire sought demographic information such as age, gender, education level and disease information such as type of cancer and stage. The type of CAM used, the reasons for use and perceived benefits were captured. Inquiries about diclosure of CAM use to physicians and the physician’s responses were also included.

### Data collection

The study population included two cohorts, Cohort 1: patients enrolled between 2006 and 2008 and Cohort 2 patients enrolled between 2016 and 2018. Convenience sampling technique was used in both cohorts. Eligible patients attending the Oncology Clinics at our center were offered to participate in the study and those who consented were enrolled in the study and completed the survey. The findings of some these individual cohorts were published previously describing the tool used for the study and results of individual cohort [12, 15].

Data from both studies were transferred into (excel database), study participants were divided into 2 cohorts based on when the data was collected. Means and proportions of the study population were calculated for study participants, overall and in groups. To determine the patients’ perception characteristics changes associated with the time change, the two cohorts were compared using Chi-squared test or Fisher exact test for categorical factors and t Students’ t-test or Mann-Whitney U Test for continuous variables as appropriate. The level of significance was declared at α = 0.05. Statistical analysis was conducted using SAS 9.4 (SAS Institute Inc., Cary, NC, USA).

## Results

### Patients’ characteristics

A total of 1416 patients were enrolled in the study in both cohorts; 464 patients in Cohort 1 and 952 patients in Cohort 2. Patients’ characteristics are detailed in Table 1.

**Table 1:** Patient Characteristics (*N* = 1416)

Characteristics	2006–2008 ***N*** = 464 N(%)	2016–2018 *N* = 952 N(%)	***P*** value*
Gender			
Male	184 (39.7)	367 (38.5)	0.6890
Female	280 (60.3)	585 (61.4)	
Median Age/Range	54 (34–72)	56 (26–81)	0.3840
Marital Status: *N* = 1408			
Married	337 (73.90)	654 (68.7)	0.0857
Single	43 (9.4)	117 (12.3)	
Separated	1 (0.2)	14 (1.5)	
Divorced	13 (3)	28 (3)	
Widow	62 (13.6)	139 (14.6)	
***Level of Education: N = 1404***			
Illiterate	51 (11.3)	100 (10.5)	<.0001
Primary	73 (16.1)	162 (17.0)	
Intermediate	188 (41.6)	280 (29.4)	
Secondary	71 (15.7)	243 (25.5)	
Higher education	69 (15.3)	167 (17.5)	
***Work Status: N = 1358***			
No Job	328 (70.7)	752 (79)	0.0006
Job	136 (29.3)	200 (21.)	
***Disease Type: N = 1416***			
Solid tumor	342 (73.7)	770 (80.9)	0.0020
Hematological malignancy	122 (26.3)	182 (19.1)	
***Treatment Type:***			
Surgery	251 (54.1)	527 (55.4)	0.6541
Radiation	79 (17.0)	417 (43.8)	<.0001
Chemotherapy	423 (91.2)	780 (81.9)	<.0001
SCT	0 (0.00)	87 (9.1)	<.0001
No treatment	21 (4.5)	44 (4.6)	0.9354

Higher education: college and postgraduate, SCT: stem cell transplantation

*The Chi-squared test statistic is significant at < 0.05

There was no statistical difference between the two cohorts in terms of age, gender, or marital status. However, Cohort 2 included more patients with higher educational levels, higher unemployment, more solid tumors, and more patients who received radiation therapy and stem cell transplant. (Table 1) The increase in the use of stem cell transplant and radiation therapy in the second cohort can be explained by the establishment of these services at our institution at 2010 and 2015, respectively at our institution and patients did not have routine access to them prior to that and were referred to outside facilities.

### Lifestyle changes and the use of complementary and alternative medicine

There were fewer patients doing exercise before and after the diagnosis of cancer, more patients who never smoke but fewer patients who quit smoking in Cohort 2 compared to Cohort 1. Furthermore, the use of CAM was significantly less in Cohort 2 (78.89%) compared to the Cohort 1 (96.8%) *p* **< .0001**. (Table 2).

**Table 2:** Changes in patients’ life styles and the use of complementary and alternative medicine

Question	Answer	Cohort 1	Cohort 2	All	*P*- Value*
Exercise before illness	No	221 (47.6)	673 (70.7)	894 (63.1)	<.0001 **
	Yes	243 (52.4)	279 (29.3)	522 (36.9)	
Exercise after illness	No	347 (74.8)	783 (82.2)	1130 (79.8)	0.0010 **
	Yes	117 (25.2)	169 (17.8)	286 (20.2)	
Smoking habit after diagnosis	Yes	20 (4.3)	64 (6.7)	84 (5.9)	0.0009 **
	Never	367 (79.1)	794 (83.4)	1161 (82.0)	
	Stop	66 (14.2)	85 (8.9)	151 (10.7)	
	Unk	11 (2.4)	9 (0.9)	20 (1.4)	
Do you use any complementary or alternative therapy?	No	15 (3.2)	201 (21.1)	216 (15.3)	<.0001 **
	Yes	449 (96.8)	751 (78.9)	1200 (84.7)	
Did you delay cancer treatment in order to CAM?	No	413 (92.0)	691 (92.0)	1104 (92.0)	0.9860
	Yes	36 (8.0)	60 (8.0)	96 (8.0)	
Patients reporting CAM-related monthly cost	N	234	626	860	<.0001 **
Approximate monthly cost (SAR)^a^	Mean	951.9 (2058.6)	429.3 (4133.4)	571.5 (3692.5)	<.0001**
Reporting CAM use to nurse	No	446 (99.3)	742 (98.8)	1188 (99.0)	0.5512
	Yes	3 (0.7)	9 (1.2)	12 (1.0)	
Reporting CAM use to the doctor	No	307 (68.4)	483 (64.3)	790 (65.8)	0.1513
	Yes	142 (31.6)	268 (35.7)	410 (34.2)	
Doctor’s response to CAM use (As reported by patient)	Opposing	26 (19.4)	131 (48.7)	157 (39.0)	<.0001 **
	Neutral	33 (24.6)	62 (23.0)	95 (23.6)	
	Supporive	75 (56.0)	76 (28.3)	151 (37.5)	

*CAM* Complementary and alternative medicine

*The Chi-squared test statistic is significant at < 0.05

^a^*SAR* Saudi Riyals (1 Saudi Riyal equals 0.27 USD)

More patients reported some CAM-related monthly cost in Cohort 2 compared to Chohort 1 (626 vs 234). However, the approximate cost of the alternative therapy per month was much higher in Cohort 1than Cohort 2 (952 vs 429 Saudi riyals per month, *p* < .0001. This translates to USD 254 vs USD 115. While this per person per month CAM-related cost, this may be a significant amount of a household income. To put into perspective, that figure may represent between 14 and 32% of a local lower-income household with a monthly income of 3000 Saudi Riyals. Reporting the use of CAM to health care staff (nurse or physician) remains low in both groups. Only 31.6% of Cohort 1 and 35.7% of Cohort 2 reported the use of CAM to their physicians, which is not significantly different (p 0.1513). More physicians opposed to the use of CAM in Cohort 2 (48.7%) compared to only 19.1% in Cohort 1, *p* < .0001. A similar percentage of patients (8%) delayed standard cancer treatment to try CAM. (Table 2) The low reporting rate to nurses compared to physician could be attributed to language barrier as most nurses are not Arabic speaking.

### Reasons for the complementary and alternative medicine use

Patients in Cohort 2 were more likely to report using CAM to control pain, improve appetite, increase strength, enhance immunity, improve mood, and for religious and social beliefs. However, fewer patients in Cohort 2 (73%) used CAM as a treatment for cancer compared to (84.4%) in Cohort 1 *p* < .0001. (Table 3).

**Table 3:** Reasons for Complementary and Alternative Medicine use

Reason for Using CAM	Response	Cohort 1	Cohort 2	ALL	*P*-Value*
To treat cancer	No	70 (15.6)	203 (27.0)	273 (22.8)	<.0001 **
	Yes	379 (84.4)	548 (73.0)	927 (77.3)	
To control pain	No	441 (98.2)	669 (89.1)	1110 (92.5)	<.0001 **
	Yes	8 (1.8)	82 (10.9)	90 (7.5)	
To improve appetite	No	447 (99.6)	712 (94.8)	1159 (96.6)	<.0001 **
	Yes	2 (0.4)	39 (5.2)	41 (3.4)	
To increase energy	No	444 (98.9)	691 (92.0)	1135 (94.6)	<.0001 **
	Yes	5 (1.1)	60 (8.0)	65 (5.4)	
To improve mood	No	445 (99.1)	589 (78.4)	1034 (86.2)	<.0001 **
	Yes	4 (0.9)	162 (21.6)	166 (13.8)	
To enhance immunity	No	442 (98.4)	655 (87.2)	1097 (91.4)	<.0001 **
	Yes	7 (1.6)	96 (12.8)	103 (8.6)	
For religious beliefs	No	439 (97.8)	636 (84.7)	1075 (89.6)	<.0001 **
	Yes	10 (2.2)	115 (15.3)	125 (10.4)	

*CAM* Complementary and alternative medicine

*The Chi-squared test statistic is significant at < 0.05

### Trends in perceived benefits of complementary and alternative medicine

More patients in Cohort 2 perceived that CAM improved pain control, appetite, and cancer response compared to Cohort 1 in which more patients believed that CAM improved their general condition. More patients in the Cohort 2 believed that the improvement was due to the medical treatment not to CAM. (Table 4).

**Table 4:** Trends in perception about the benefits of complementary and alternative medicine benefits

Perception	Response	Cohort 1	Cohort 2	All	*P*-value*
Decreasing pain	No	421 (93.8)	635 (84.6)	1056 (88.0)	<.0001 **
	Yes	28 (6.2)	116 (15.4)	144 (12.0)	
Improved appetite	No	438 (97.6)	651 (86.7)	1089 (90.8)	<.0001 **
	Yes	11 (2.4)	100 (13.3)	111 (9.3)	
Improvement in general condition	No	126 (28.1)	438 (58.3)	564 (47.0)	<.0001 **
	Yes	323 (71.9)	313 (41.7)	636 (53.0)	
Reason for improvement CAM Use	No	372 (82.9)	720 (95.9)	1092 (91.0)	<.0001 **
	Yes	77 (17.1)	31 (4.1)	108 (9.0)	
Reason for improvement- Medical Treatment	No	415 (92.4)	530 (70.6)	945 (78.8)	<.0001 **
	Yes	34 (7.6)	221 (29.4)	255 (21.3)	
Reason for improvement Both CAM and Medical Treatment	No	165 (36.7)	283 (37.7)	448 (37.3)	0.7460 **
	Yes	284 (63.3)	468 (62.3)	752 (62.7)	

*CAM* complementary and alternative medicine

*The Chi-squared test statistic is significant at < 0.05

### Trends in specific complementary and alternative medicine use

More patients used herbal mixture, Zamzam water (A holy water found only in Mecca, Saudi Arabia), camel milk, garlic, and multivitamins as well as other herbs and supplication in Cohort 2 compared to Cohort 1 and fewer people used black seeds (*Nigella sativa*). (Table 5).

**Table 5:** Trends in specific complementary and alternative medicine use

CAM Type	Response	Cohort 1	Cohort 2	ALL	*P*-Value*
Zamzam water	No	446 (99.3)	207 (27.6)	653 (54.4)	<.0001 **
	Yes	3 (0.7)	544 (72.4)	547 (45.6)	
Black seed	No	185 (41.2)	535 (71.2)	720 (60.0)	<.0001 **
	Yes	264 (58.8)	216 (28.8)	480 (40.0)	
Camel milk	No	398 (88.6)	611 (81.4)	1009 (84.1)	0.0008 **
	Yes	51 (11.4)	140 (18.6)	191 (15.9)	
Camel urine	No	410 (91.3)	665 (88.5)	1075 (89.6)	0.1291 **
	Yes	39 (8.7)	86 (11.5)	125 (10.4)	
Garlic	No	393 (87.5)	572 (76.2)	965 (80.4)	<.0001 **
	Yes	56 (12.5)	179 (23.8)	235 (19.6)	
Olive oil	No	229 (51.0)	451 (60.1)	680 (56.7)	0.0022 **
	Yes	220 (49.0)	300 (39.9)	520 (43.3)	
Multivitamins	No	442 (98.4)	641 (85.4)	1083 (90.3)	<.0001 **
	Yes	7 (1.6)	110 (14.6)	117 (9.8)	
Quran Recitation	No	87 (19.4)	163 (21.7)	250 (20.8)	0.3366 **
	Yes	362 (80.6)	588 (78.3)	950 (79.2)	
Supplication	No	420 (93.5)	138 (18.4)	558 (46.5)	<.0001 **
	Yes	29 (6.5)	613 (81.6)	642 (53.5)	

*The Chi-squared test statistic is significant at < 0.05

*CAM* Complementary and alternative medicine. Zamzam water: A holy water found only in Mecca, Saudi Arabia

There were no differences in the percentage of patients who practiced the Quran recitation in both cohorts.

## Discussion

Our study revealed very interesting trends in CAM use by cancer patients as well as their perception about the benefits of CAM. Although the study revealed a reduction in the overall CAM use, there are very noticeable changes in the trend that are worth paying attention to. This trend of reduction in using CAM maybe due to more physician opposing the use of CAM as well as increased awareness of both of the public and the healthcare professionals of the potential harm of some of the CAM, due to interaction with the medications or the inherent side effects of CAM or avoiding getting treatment [16–18].

In a serial cross sectional study of 43,644 patients between 1999 and 2014, cancer patients were more likely to use botanical dietary supplements (BDS) compared to others and the overall trend for the whole group was stable, however, the use of BDS declined in certain subgroups such as elderly patients and those with low income and low educational level [19]. Another earlier study showed an increase in the use of CAM among patients with breast cancer [20].

The reason for using CAM to treat cancer decreased significantly with more confidence that the overall improvement in patients’ condition is due to the medical treatment. However, there was a trend to use CAM to manage symptoms such as pain, lack of appetite, fatigue and emotional and mood disturbance; which highlights the importance of the timely implementation of supportive care. The patients use these alternative therapies because they may feel that the usual medical care is not taking care of these symptoms. Interestingly, the impact of social factors and religious beliefs as reasons to use CAM was more apparent in Cohort 2 compared to the Cohort 1 [6, 21].

The perception of CAM benefits in controlling the pain and improving the appetite and contributing to the cancer response was apparent in the second cohort compared to the first. Cohort 1 patients perceived more improvement in their condition which was attributed to CAM, unlike Cohort 2 patients who were more likely to believe that the improvement was due to medical treatment and not to CAM. The trend to have less use of CAM in general and the less CAM use to treat cancer, in particular, may reflect the better education of the patients as well as the social and cultural changes in the society.

The trend in the specific types of CAM used showed that herbal mixture is being used more in the second cohort, however, alternative therapies of religious nature increased significantly such as the use of Zamzam water or water with Quran recited on it. The use of supplication increased significantly, while Quran recitation did not change. Black seeds consumption was less in the second cohort as well as olive oil. However, there was an increase in the use of multivitamins, garlic and camel milk. This reflects the continued impact of religious background on these practices because camel milk, Zamzam water and the water with the Quran recited on it are all of the religious backgrounds.

The use of herbal and plant products such as olive oil and black seeds originate from religious background and reported to be used by Muslim patients in different countries especially in Saudi Arabia. The use of these products may reach up to 68% of the patients. Olive oil can be ingested or used externally by rubbing it on the skin [22–25].

Zamzam water is a holy water that is found in the Holy city of Mecca and used based of the belief in its healing and spiritual properties and its ingestion was reported by authors from different countries [26–28].

The level of exercise before the illness or after the illness is much less in the second cohort. In addition, more people smoked in the second cohort with less likelihood to quit after diagnosis compared to the first cohort. There is a considerable number of patients delaying their cancer treatment to give a chance for the CAM to work. This may expose the patients to harm from disease progression or complication that deserves further investigation and implementation of remedial measures.

Interestingly, the majority of patients do not divulge to their physicians their use of CAM. In a systemic review of 21 studies, 20–90% of the patients did not disclose their use of CAM to the providers for many reasons, including lack of inquiry from physicians or anticipation of doctors disapproval among others [29]. Interestingly, in our study, more physicians seem to be opposing to CAM use with the second cohort. The low reporting rate highlights the need to educate the patients about the importance of disclosing to and encouraging the healthcare team to systematically inquire about the use of CAM in a very proactive way and not to count on the voluntary reports of the patient.

Although our study revealed significant change in the prevalence and pattern of CAM use over a decade; however, the reasons for these changes were not addressed in our study especially the impact of social media and the cultural transformation of the whole society. There are multiple factors, which might have contributed to these changes, such as the different levels of education and the type of cancer and treatment received. The fact that the second cohort are more educated with more solid malignancies and receiving more radiation therapy and stem cell transplant can not be ignored. However, these cannot be taken in isolation from the larger societal changes. Further studies are needed to investigate the reasons that prevented non-CAM users from doing so.

## Conclusion

Although there was a reduction in its use, majority of patients still using some kind of CAM. The reasons for the use require further investigation as it may reflect gaps in the healthcare delivery which is not addressing all patients needs. Disclosure of CAM use for healthcare professionals remains a concern that need to be addressed systematically.

## Data Availability

Data and material are available.

## Abbreviations

CAM: Complementary Alternative Medicine
NCCAM: National Centre for Complementary and Alternative Medicine
BDS: Botanical dietary supplements
